# Recurrent Ileo-ileal Intussusception Caused by Inflammatory Fibroid Polyp: A Rare Case Report

**DOI:** 10.34172/aim.2023.53

**Published:** 2023-06-01

**Authors:** Gökmen Güzel, Muhammer Ergenç

**Affiliations:** ^1^Department of General Surgery, Serik State Hospital, Antalya, Turkey; ^2^Department of General Surgery, Istanbul Sultanbeyli State Hospital, Istanbul, Turkey

**Keywords:** Intestinal obstruction, Invagination, Small intestine

## Abstract

An inflammatory fibroid polyp is a rare benign lesion of the gastrointestinal tract, which can cause obstruction or intussusception when it reaches a large diameter. We present a case of a 46-year-old female admitted to our clinic with recurrent ileus attacks. We performed segmental resection of the small bowel due to a 3-cm pedunculated polypoid lesion located in the terminal ileum that caused ileo-ileal intussusception and whose pathology was reported as an inflammatory fibroid polyp. In adults presenting with ileus, the possibility of intussusception should be kept in mind.

## Introduction

 Intussusception is the invagination of one part of the intestine into another segment; it is rarely seen in adults. Invagination is mostly seen in the pediatric age group, the clinical manifestation emerges rapidly, and its etiology is idiopathic.^1^ Adults typically have a more chronic history of illness; 90% of the intussusceptions are caused by a lesion, and there is the possibility of malignancy. Ischemia, necrosis, perforation, and obstruction may develop in the invaginated segment.^2-4^

 An inflammatory fibroid polyp is a rare benign lesion of the gastrointestinal tract. It is most commonly seen in the gastric antrum, followed by the ileum and the colon in decreasing order of frequency. It is primarily asymptomatic and is incidentally detected unless it causes complications (such as bleeding, perforation, or ileus) like other small bowel tumors. When it reaches a large diameter, it can cause obstruction or intussusception.^5,6^

 We performed segmental ileum resection in a case of inflammatory fibroid polyp causing recurrent ileus attacks and ileo-ileal intussusception. By presenting our case, we aim to remind the reader that the etiology of adult intussusception is mainly tumoral lesions.

## Case Presentation

 A 46-year-old female patient was admitted to the emergency department of our hospital with complaints of abdominal pain lasting for a week, nausea, occasional vomiting, and inability to defecate for four days. When the patient was questioned, we learned that she was followed up in another hospital for three days about four months ago with similar complaints and was discharged with the recommendation of an elective colonoscopy.

**Figure 1:**
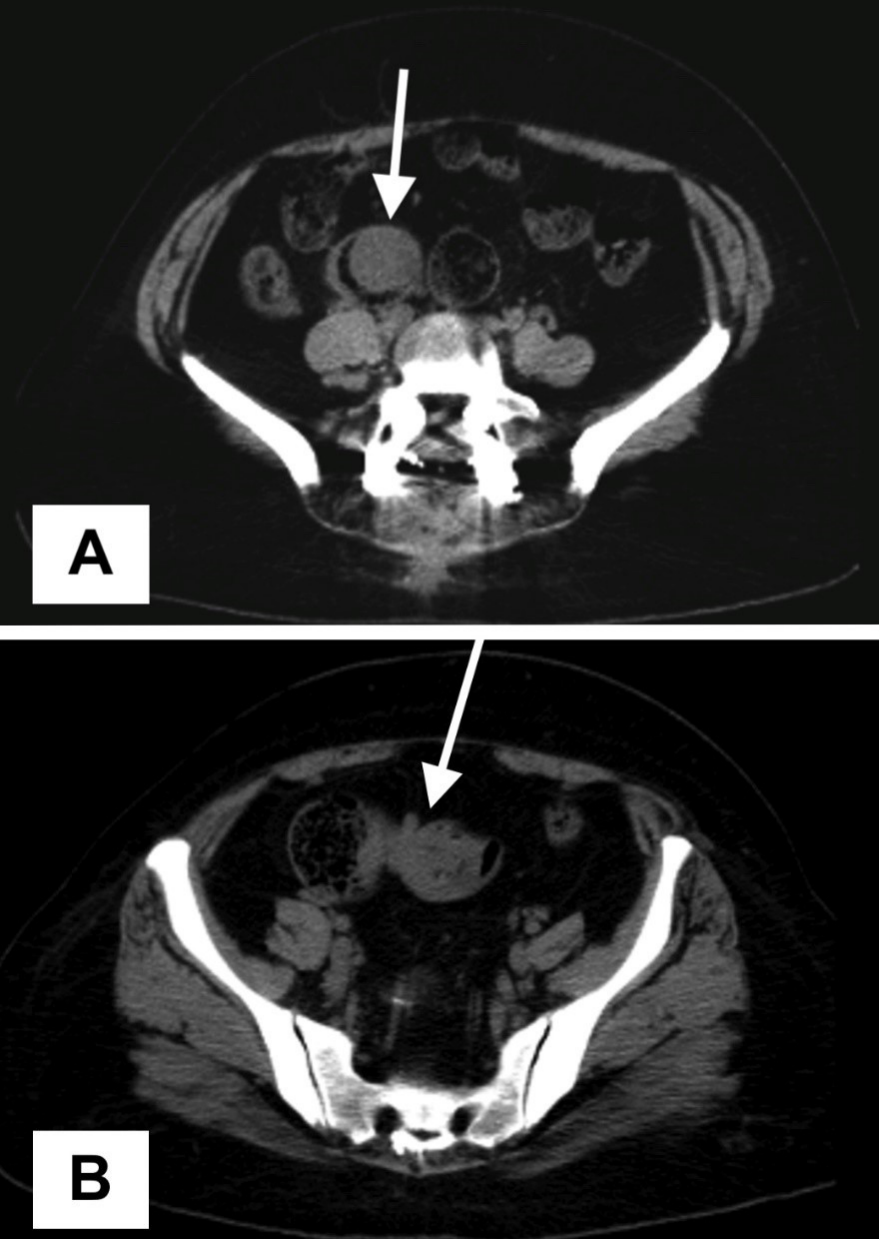
Abdominal Computed Tomography Images. (A) The polypoid lesion with a diameter larger than two cm, protruding from the wall to the lumen in the terminal ileum (white arrow). (B) A nested segment which is compatible with ileo-ileal intussusception (white arrow)

 In the abdominal examination of the patient, distension and tenderness in the lower quadrants were detected. There was no defense or rebound. Bowel sounds could not be heard. No pathology was found on rectal examination. The patient had no history of abdominal surgery and was followed for a long time due to Behçet’s disease. The patient’s white blood cell count was 14 × 10^3^/µL, C-reactive protein was 36 mg/L, and other biochemical parameters were normal. Erect abdominal radiography revealed several air-fluid levels at the small intestine level. On abdominal ultrasonography, an intraluminal hypoechoic nodular lesion with a diameter of 47 mm (suspicious intraluminal pathology in the cecum), distension in the adjacent terminal ileum, and thickening of the intestinal walls were observed. Also, according to the abdominal computed tomography (CT) taken without contrast, a nodular lesion larger than two cm in diameter and soft tissue density extending from the wall to the lumen in the terminal ileum (suspected polyp) was observed in the sections passing through the level of the pelvic inlet (Figure 1A) together with a nested segment appearance (proximal to the described polypoid lesion) compatible with ileo-ileal intussusception (Figure 1B).

**Figure 2:**
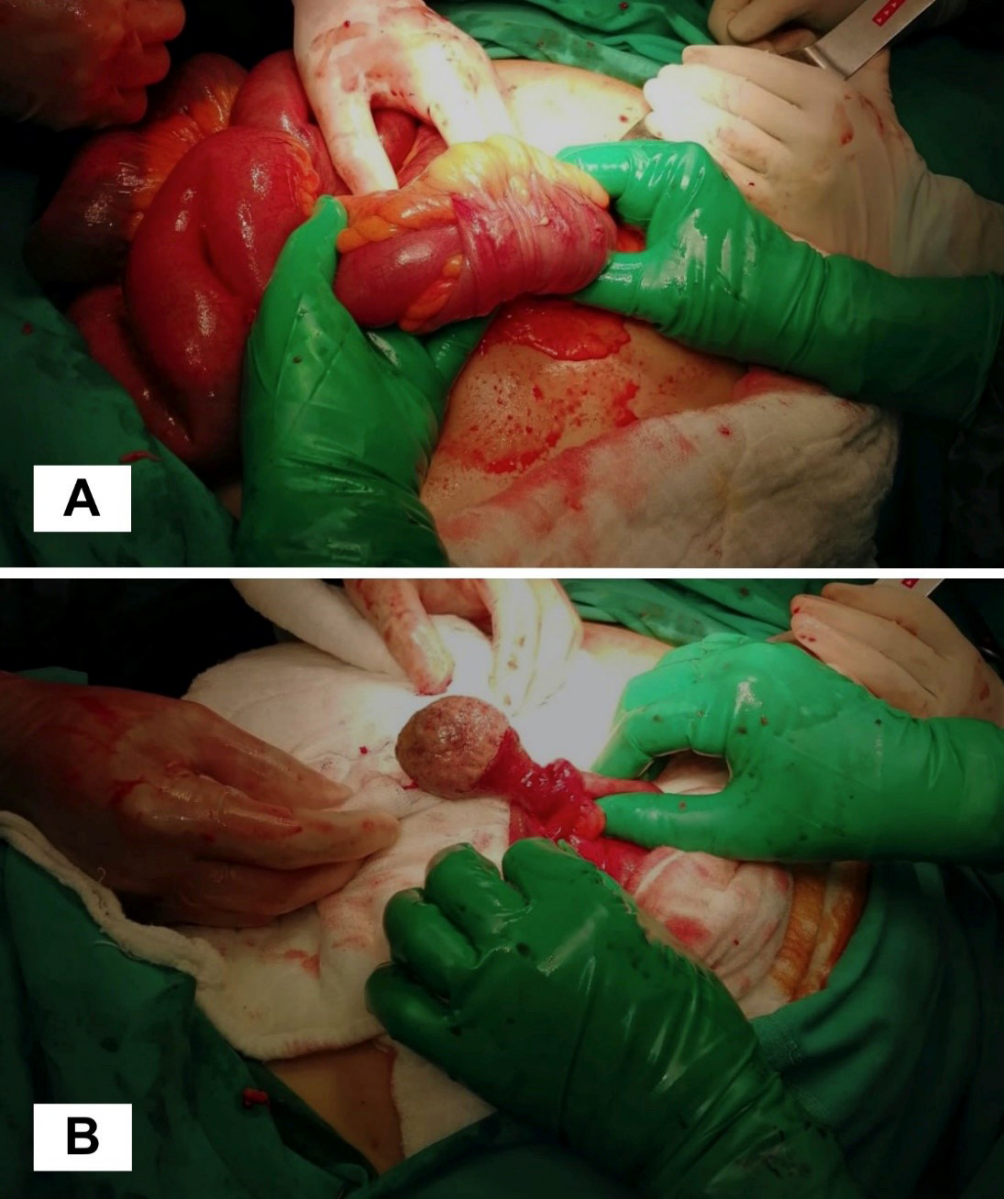
(A) Intraoperative image of ileo-ileal invagination. (B) Intraoperative image of the pedunculated polypoid lesion detected after enterotomy following reduction of intussusception

 The patient underwent emergency surgery. In intraoperative examination, a 10-cm long ileo-ileal intussusception area was detected at a distance of approximately 15 cm from the ileocecal valve (Figure 2A). A rubbery solid mass filling the lumen was noticeable when the invaginated segment was palpated. When the lumen was entered after the reduction of the invagination, a benign polypoid lesion was found with a diameter of about three cm with a stalk; the length of the stalk was two cm, and the diameter was nearly 1.5 cm (Figure 2B). The invaginated segment was hyperemic and partially edematous. Small bowel anastomosis was performed after segmentary resection involving the intussusception area and the polypoid lesion. The patient was discharged on the sixth postoperative day with full recovery. The specimen’s pathology was reported as an inflammatory fibroid polyp.

## Discussion

 The incidence, causes, and treatment modalities of intussusception in the pediatric and adult age groups are very different. Non-surgical treatment methods are preferred in cases of intussusception in the pediatric age group.^1,2,6^ One to three percent of ileus cases requiring surgery in adults are due to intussusception.^7^ A solitary pathology mostly causes intussusception in the adult age group.

 The clinical manifestation may be acute, intermittent, or chronic. Intussusception can also be entero-enteral, ileocecal, ileocolic, or colo-colonic, depending on the localization. The severity and form of the complaints vary according to the location of the invagination.^2,8^ Although the diagnosis is typically made by surgery, the diagnostic value of abdominal CT prior to surgery is extremely high. The possibility of underlying malignant pathology in colo-colonic intussusceptions is relatively higher.^8^ Preoperative colonoscopy can also be performed in case of colonic intussusception. In entero-enteral invaginations, it is more likely that the cause is a benign lesion. Intestinal invaginations mainly originate from the small bowel.^3^ Among the pathologies that may cause small bowel intussusception are hamartoma, leiomyoma, adenoma, Meckel’s diverticulum, polyps due to Peutz-Jeghers syndrome, and adhesions arising from previous abdominal surgeries.^2,9,10^ Malignant pathologies (adenocarcinoma) that cause intussusception in the small intestine are seen less frequently than benign lesions.^1^

 An inflammatory fibroid polyp is a very rare benign pathology that causes intussusception at the small intestine level.^5,6^ Treatment of intussusception in adults is determined according to the etiology. If intussusception is due to adhesions, it is sufficient to eliminate the adhesions and mechanical problems. In other pathologies, reduction alone is not enough.^10^ Resection of the affected bowel segments is recommended in the presence of ischemia, inflammation, and colonic intussusception.^1-3,10^ After reduction, excision of the benign-appearing lesion by enterotomy is an option for intussusception if there is no ischemia and inflammation in the small intestine.^2^

 In our case, we preferred segmental resection because the base area of the stem part of the pedunculated polypoid lesion was also quite large, and the ileal loops were edematous and hyperemic.

 In adults presenting with ileus, the possibility of intussusception should be kept in mind, and abdominal computed tomography should be performed whenever possible and carefully examined. When invagination is detected, the reduction should not be contented with; even if there is no external pathology, the proximal and distal parts of the invaginated segments should be palpated to avoid missing possible intraluminal pathologies. While applying surgical treatment, the choice should be made according to the determined etiology.
